# Probiotic Diversity Enhances Rhizosphere Microbiome Function and Plant Disease Suppression

**DOI:** 10.1128/mBio.01790-16

**Published:** 2016-12-13

**Authors:** Jie Hu, Zhong Wei, Ville-Petri Friman, Shao-hua Gu, Xiao-fang Wang, Nico Eisenhauer, Tian-jie Yang, Jing Ma, Qi-rong Shen, Yang-chun Xu, Alexandre Jousset

**Affiliations:** a Jiangsu Provincial Key Lab for Organic Solid Waste Utilization, National Engineering Research Center for Organic-based Fertilizers, Jiangsu Collaborative Innovation Center for Solid Organic Waste Resource Utilization, Nanjing Agricultural University, Nanjing, People’s Republic of China; b Utrecht University, Institute for Environmental Biology, Ecology and Biodiversity, Utrecht, the Netherlands; c Department of Biology, University of York, Wentworth Way, York, United Kingdom; d German Centre for Integrative Biodiversity Research (iDiv) Halle-Jena-Leipzig, Leipzig, Germany; e Leipzig University, Institute of Biology, Leipzig, Germany

## Abstract

Bacterial communities associated with plant roots play an important role in the suppression of soil-borne pathogens, and multispecies probiotic consortia may enhance disease suppression efficacy. Here we introduced defined *Pseudomonas* species consortia into naturally complex microbial communities and measured the importance of *Pseudomonas* community diversity for their survival and the suppression of the bacterial plant pathogen *Ralstonia solanacearum* in the tomato rhizosphere microbiome. The survival of introduced *Pseudomonas* consortia increased with increasing diversity. Further, high *Pseudomonas* diversity reduced pathogen density in the rhizosphere and decreased the disease incidence due to both intensified resource competition and interference with the pathogen. These results provide novel mechanistic insights into elevated pathogen suppression by diverse probiotic consortia in naturally diverse plant rhizospheres. Ecologically based community assembly rules could thus play a key role in engineering functionally reliable microbiome applications.

## IMPORTANCE

The increasing demand for food supply requires more-efficient control of plant diseases. The use of probiotics, i.e., naturally occurring bacterial antagonists and competitors that suppress pathogens, has recently reemerged as a promising alternative to agrochemical use. It is, however, still unclear how many and which strains we should choose for constructing effective probiotic consortia. Here we present a general ecological framework for assembling effective probiotic communities based on *in vitro* characterization of community functioning. Specifically, we show that increasing the diversity of probiotic consortia enhances community survival in the naturally diverse rhizosphere microbiome, leading to increased pathogen suppression via intensified resource competition and interference with the pathogen. We propose that these ecological guidelines can be put to the test in microbiome engineering more widely in the future.

## INTRODUCTION

Biodiversity-ecosystem functioning (BEF) experiments suggest that species diversity provides various community-level benefits related to productivity (1, 2), cycling of nutrients, rates of decomposition, resistance to environmental change, and resistance to species invasions. Such relationships are omnipresent and, in the case of microbes, play an important role also in the health of higher organisms by ensuring efficient functioning of the host-associated microbiome (3). In the case of plant-microbe interactions, high bacterial diversity has been associated with increased resistance to pathogen invasions and plant infestation (2, 3), for example, via intensified resource competition (4–6). Several studies have also shown that community composition and diversity can affect the invasion/colonization success of additional species (4–6). Here we studied the potential beneficial effects of microbial diversity in the context of probiotic bacterial community performance. We hypothesized that diversity could affect the establishment, survival, and functioning of introduced microbial consortia in the complex plant microbiome and could shape the ability of the community to induce disease suppression.

Biodiversity effects could drive the functionality of introduced rhizosphere bacterial communities in different ways (7). First, high levels of species richness can increase the total number of resources that species can collectively utilize as a community (niche breadth) (5). This could improve community survival in the temporally and spatially fluctuating rhizosphere environment and ensure that at least one of the species will survive under the prevailing conditions (8). Wide community niche breadth is also expected to intensify resource use in general, which could help bacteria to better colonize and persist in the rhizosphere (9, 10). Furthermore, wide niche breadth is likely to intensify the resource competition between the introduced bacterial community and a potential pathogen, which could lead to competitive exclusion of the pathogen (5, 11) and, in the present context, to elevated host plant protection.

Biodiversity of the introduced rhizosphere bacterial communities could also affect interference competition with other microorganisms, including both the resident microbiota and pathogens. For example, previous studies have shown that the production of secondary metabolites that suppress pathogen growth (12, 13) can increase with the density and richness of the inoculated probiotic consortia (14, 15). As a result, diverse bacterial communities could be more effective at suppressing invading pathogens. Similarly, secondary metabolites may help the introduced microbial communities to compete with the indigenous microbiota, enhancing their survival. Furthermore, a combination of different bacterial secondary metabolites produced jointly by a diverse community could result in stronger antagonism toward the pathogen if they target different cellular functions (16)—an idea analogous to mixing antibiotics from several antibiotic classes to achieve higher pathogen inhibition (and reduced resistance evolution) in clinical environments (17). The interplay between bacterial strains in diverse bacterial communities may also involve species-specific responses that trigger complex secretion systems leading to induction or upregulation of secondary metabolites or signal molecules that inhibit pathogen growth (18). Surprisingly, despite a growing interest in using microbial consortia in plant protection, there have been hardly any studies investigating how the diversity and composition of introduced probiotic consortia may affect their functioning.

Here we used complementary laboratory and greenhouse experiments to study the mechanisms and importance of biodiversity of introduced plant growth-promoting *Pseudomonas* species communities for disease suppression within the natural rhizosphere microbiome. Eight *Pseudomonas* species strains producing the broad-spectrum antibiotic 2,4-diacetylphloroglucinol (DAPG) were used in this study. We assembled *Pseudomonas* communities at four richness levels as described previously (19, 20). We chose *Pseudomonas* bacteria due to their well-reported disease suppression abilities and widespread occurrence in the rhizosphere (12, 21). We first used simple *in vitro* experiments to quantify the relationship between *Pseudomonas* community strain richness and composition and traits linked to resource competition and antagonism. In order to bridge the gap between the laboratory and the real world, we then assessed the ability of different *Pseudomonas* communities to survive *in vivo* in the naturally highly diverse tomato plant rhizosphere (homogenized natural soil) and to suppress the growth of the *Ralstonia solanacearum* bacterial pathogen—the causative agent of global bacterial wilt disease epidemics (22). We found that high biodiversity enabled the introduced *Pseudomonas* community to persist at high density in the rhizosphere throughout the experiment, leading to dramatically increased pathogen suppression and lower disease incidence. These patterns matched well with the *in vitro* results: increasing *Pseudomonas* community diversity increased the intensity of both resource and interference competition, which in turn resulted in very low pathogen densities. Together, these results suggest that BEF and competition theory could thus provide community assembly rules for engineering functionally reliable microbiome applications.

## RESULTS

### BEF relationships *in vitro*.

**FIG 1:**
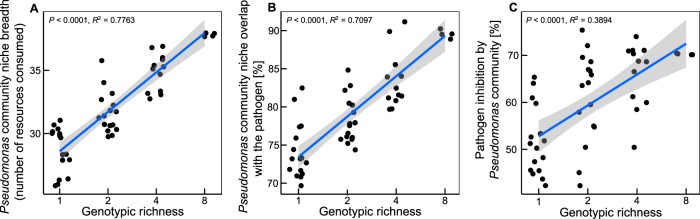
Characterization of biodiversity-ecosystem functioning relationships *in vitro*. (A) *Pseudomonas* community niche breadth was defined as the number of carbon sources used by at least one of the members of *Pseudomonas* community (detailed information on resources can be found in Table S4). (B) *Pseudomonas* community niche overlap with the pathogen was defined as similarity in resource consumption between the resident community and the pathogen. (C) Antibacterial activity of *Pseudomonas* community was determined as a reduction in pathogen density in the presence of *Pseudomonas* bacterial supernatants; all supernatants were derived from monocultures and mixed together in testing the synergistic effects.

Increasing *Pseudomonas* community genotypic richness correlated positively with community niche breadth (*R*^2^ = 0.776, *P* < 0.0001, Fig. 1A), overlapping of the niche with the pathogen (*R*^2^ = 0.709, *P* < 0.0001, Fig. 1B), and direct pathogen inhibition (*R*^2^ = 0.389, *P* < 0.0001, Fig. 1C) *in vitro*.

### BEF relationships *in vivo*.

**FIG 2:**
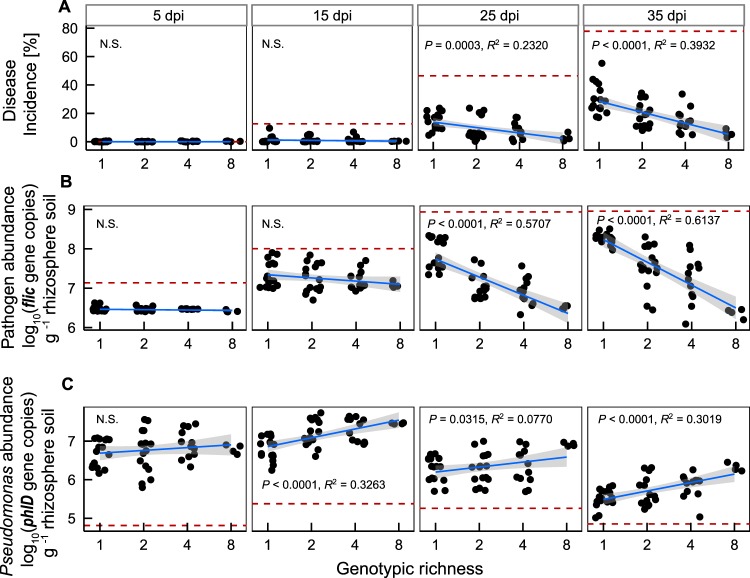
Characterization of biodiversity-ecosystem functioning relationships *in vivo*. (A) The dynamics of bacterial wilt disease incidence in *Pseudomonas* communities at different richness levels and at different points in time. (B) Pathogen density dynamics as affected by *Pseudomonas* communities with different richness levels. (C) *Pseudomonas* density dynamics in communities with different richness levels. Panel columns denote results at 5 days, 15 days, 25 days, and 35 days post-pathogen inoculation (dpi). The red dashed lines show the baseline for control treatments. In panels A and B, red dotted lines denote disease incidence and pathogen density in the absence of *Pseudomonas* bacteria; in panel C, red dashed lines denote *Pseudomonas*-specific *phlD* gene density in natural soil in the absence of introduced *Pseudomonas* bacteria.

Both disease incidence and pathogen density decreased significantly with increasing *Pseudomonas* community richness (Fig. 2A and B and Table 1). While all *Pseudomonas* monocultures reduced disease incidence to some extent, they offered only partial protection against bacterial wilt disease. In contrast, the 8-strain community provided almost complete protection against bacterial wilt, and 2- and 4-strain communities provided intermediate levels of protection (Fig. 2A and Table 1). The effect of *Pseudomonas* community richness on disease suppression increased with time (Fig. 2B, significant richness × time interaction, and Table 1): while community richness had no effect on disease suppression during the first 15 days after pathogen invasion, the 8-strain *Pseudomonas* community reduced pathogen density by 99% compared to the best performing monoculture on day 35 (Fig. 2B and Table 1).

**TABLE 1:** ANOVA table on the main and interactive effects of genotypic richness and time on bacterial wilt incidence (proportion of wilted plants) and pathogen and probiotic *Pseudomonas* community abundances in the rhizosphere^a^

Parameter(s)	Disease incidence			Pathogen abundance		*Pseudomonas* abundance	
	*df*	*F* (*R*^2^: 0.64)	*P* (AIC: 30.6)	*F* (*R*^2^: 0.62)	*P* (AIC: 195.7)	*F* (*R*^2^: 0.51)	*P* (AIC: 248.7)
Richness	1	30.6	<0.0001	74.6	<0.0001	21.7	<0.0001
Time	1	275.6	<0.0001	181.2	<0.0001	175.0	<0.0001
Richness × time	1	30.8	0.0002	52.3	<0.0001	2.3	0.1305
No. of residuals	188						

a All response variables were treated as continuous variables, and the genotypic richness and *Pseudomonas* abundance data were log-transformed before the analysis was performed. The *df* data denote degrees of freedom, *R*^2^ data denote total variance explained by the regression coefficient of determination, and AIC data denote Akaike’s information criterion. ANOVA, analysis of variance.

At the initial stage, all *Pseudomonas* communities were able to colonize plant roots equally well regardless of the community diversity. However, only the 8-strain *Pseudomonas* communities were able to maintain high population densities in the rhizosphere throughout the whole experiment (Fig. 2C, significant richness × time interaction, and Table 1), reaching densities ca. 10 times higher that those seen with the most productive single-strain community at the end of the experiment (indicative of transgressive overyielding [23]) (see Table S1 in the supplemental material). Interestingly, none of the *Pseudomonas* strains showed a particularly strong identity effect on pathogen suppression (Table S1). This suggests that high *Pseudomonas* community richness increased its ability to colonize the rhizosphere microbiome due to synergistic effects between community members instead of inclusion of one particularly efficiently colonizing *Pseudomonas* strain.

### Linking community performance *in vivo* to characteristics *in vitro*.

**FIG 3:**
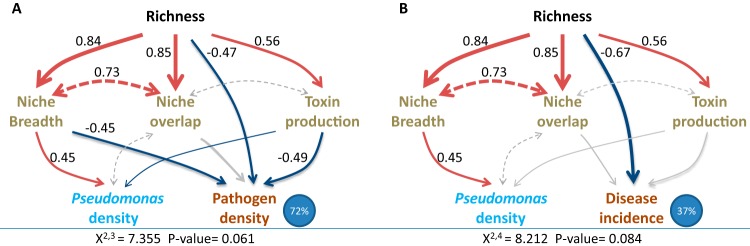
Structural equation models testing the mechanistic links between *Pseudomonas* community richness and pathogen density (A) and disease incidence (B) 35 days after pathogen inoculation. (A) Direct and indirect (corresponding to *Pseudomonas* community niche breadth and *Pseudomonas* community toxicity, respectively) richness effects on pathogen density. (B) Disease incidence data were explained only by a direct richness effect. Blue circles in both panels denote the proportion of the total variance explained. Blue arrows indicate negative relationships, and red arrows indicate positive relationships; double-headed, dashed arrows indicate undirected correlations between different variables (no hypothesis tested); and gray arrows indicate nonsignificant relationships between different variables. Arrow widths indicate the relative sizes of the effects, and the numbers beside the arrows show standardized correlation coefficients (relative effect sizes of nonsignificant correlations are not shown).

We found that *Pseudomonas* community survival in the rhizosphere increased with the increasing niche breadth of the community, while pathogen density correlated negatively with the increasing inhibition activity of *Pseudomonas* communities measured *in vitro* (Table 2). Pathogen invasion success in the rhizosphere depended also on the density of the *Pseudomonas* community (Table 2). We used a structural equation modeling (SEM) approach to further study the relative levels of importance of different mechanisms linking *Pseudomonas* community composition to disease suppression. The final models fit the data well (both *P* > 0.05) and explained 72% of the variance in pathogen density and 37% of the variance in disease incidence at day 35 of the experiment (Fig. 3). Pathogen density decreased with *in vitro* antagonistic activity against the pathogen, higher strain richness, and wider niche breadth of the *Pseudomonas* communities. Accordingly, disease incidence decreased with increasing richness of the *Pseudomonas* communities.

**TABLE 2:** ANOVA table on the effects of probiotic *Pseudomonas* community resource use with respect to niche breadth and niche overlap with the pathogen and direct pathogen inhibition (toxicity) on bacterial wilt incidence (proportion of wilted plants) and pathogen and probiotic *Pseudomonas* community abundances in the rhizosphere^a^

Parameter	Value(s)											
	5 dpi			15 dpi			25 dpi			35 dpi		
	*df*	*F*	*P*	*df*	*F*	*P*	*df*	*F*	*P*	*df*	*F*	*P*
Disease incidence												
Toxicity		NR	NR		NR	NR	1	7.9	0.0071	1	11.3	0.0016
NB		NR	NR		NR	NR		NR	NR	1	4.5	0.0389
NOI		NR	NR		NR	NR		NR	NR		NR	NR
No. of residuals	NR			47			46			45		
Model summary		NR			NR			*R*^2^: 0.13			*R*^2^: 0.23	
								AIC: 16.8			AIC: 55.9	
Pathogen abundance												
Toxicity	1	6.6	0.0135	1	4.7	0.0358	1	38.4	0.0001	1	69.2	0.0001
NB		NR	NR		NR	NR		22.9	0.0001	1	17.7	0.0001
NOI		NR	NR		NR	NR		NR	NR		NR	NR
No. of residuals	46			46			45			45		
Model summary		*R*^2^: 0.11			*R*^2^: 0.07			*R*^2^: 0.56			*R*^2^: 0.64	
		AIC: −155.7			AIC: 25.4			AIC: 49.9			AIC: 58.2	
*Pseudomonas* abundance												
Toxicity		NR	NR	1	12.7	0.0009	1	4.4	0.0413		NR	NR
NB		NR	NR	1	5.6	0.0227		NR	NR	1	16.4	0.0002
NOI		NR	NR		NR	NR		NR	NR		NR	NR
No. of residuals	46			44			45			45		
Model summary		NR			*R*^2^: 0.26			*R*^2^: 0.07			*R*^2^: 0.25	
					AIC: 31.6			AIC: 49.3			AIC: 32.3	

a All response variables were treated as continuous variables, and bacterial abundances were log-transformed before the analysis. Separate models were run for each dependent variable at different time points (5, 15, 25, and 35 days post-pathogen inoculation [dpi]). Table data represent only the most parsimonious models based on the Akaike’s information criterion (AIC) where NR data denote variables that were not retained in the final models, *df* data denote degrees of freedom, and *R*^2^ data denote total variance explained by regression coefficient of determination. NB, niche breadth; NOI, niche overlap with the pathogen.

## DISCUSSION

Host-associated microbiomes play an essential role in preventing diseases (24, 25). It is still, however, less clear how to manipulate and improve the functioning of host-associated microbiomes. While microbial diversity is known to enhance community resistance to pathogen invasions in general, BEF relationships are very variable (5, 19, 26). We thus need to rethink what kind of guidelines to use for selecting species or strains that work together best in performing a desired community-level function. Here we show that amending complex rhizosphere microbiomes with carefully selected bacterial consortia based on microbial competitive interactions can improve key functions such as pathogen suppression. To this end, we used a combination of experiments to study how the diversity affected the survival and functioning of probiotic bacteria in a naturally diverse tomato rhizosphere microbiome. Only the most diverse probiotic *Pseudomonas* communities (composed of 8 strains) were able to maintain high densities in the rhizosphere throughout the experiment, and the pathogen densities correlated negatively with both density and diversity of *Pseudomonas*. The beneficial biodiversity effects on pathogen suppression could be explained via a two-step process where high *Pseudomonas* community diversity first improved the establishment and survival of the introduced probiotic community in the rhizosphere, which in turn ensured effective pathogen suppression at the later stages of infection. The positive relationship between *Pseudomonas* community diversity and the intensity of interference and resource competition thus likely helped the introduced community to compete with both nonpathogenic naturally occurring bacteria and the pathogen during the greenhouse experiment.

We found that increasing diversity increased both the number of resources that the *Pseudomonas* community was able to use for its growth and the number of resources that were also used by the pathogen (niche overlap). While all of the *Pseudomonas* communities showed comparable levels of survival in the rhizosphere during the first 2 weeks of the experiment, only the most diverse *Pseudomonas* communities were able to persist at high densities and to efficiently constrain pathogen invasion during the greenhouse experiment. One likely explanation for this is that only the diverse *Pseudomonas* communities were able to efficiently compete for resources with the pathogen and the already-present natural bacterial communities. For example, plant-derived resources may have been readily available in the rhizosphere at the beginning of the experiment, allowing introduced *Pseudomonas* strains to reach high densities regardless of their diversity. However, increases in levels of the pathogen and commensal bacteria could have intensified the resource competition toward the end of the experiment, leading to declines in *Pseudomonas* densities. These results suggest that the beneficial effect of the high diversity of the introduced *Pseudomonas* community was likely due to improved survival in the presence of competitors (9, 10).

High probiotic community diversity could have also contributed to direct inhibition of the invading pathogen by stimulating secondary metabolite production (27). In support for this, we found that mixing *Pseudomonas* supernatants from different monocultures increased pathogen suppression *in vitro*. This suggests that secondary metabolites produced by different *Pseudomonas* strains can synergistically suppress the pathogen. *Pseudomonas* bacteria produce a distinct set of secondary metabolites, including polyketides, cyanide, lipopeptides, and exoenzymes, and all of these compounds differ in their molecular mechanisms and modes of action. Diverse *Pseudomonas* communities could thus produce a higher variety of toxins that could increase the total antibacterial activity of the *Pseudomonas* community. Increased pathogen inhibition also correlated positively with the *Pseudomonas* community survival in the rhizosphere, which suggests that more-diverse communities could have exhibited elevated pathogen inhibition via density effects (the higher the *Pseudomonas* population density, the higher the amount of toxins produced). It should be noted that we did not quantify the antibacterial substances produced by *Pseudomonas* bacteria in our *in vitro* assay and, hence, that further comparative genomics and/or metabolomics approaches are needed to unravel the mechanism underlying the toxicity of *Pseudomonas*. However, the filtration technique used in our assays is fast to perform and does not require prior knowledge of the molecular nature of the secreted compounds. Hence, this method could be generalized to other taxa and could represent a valuable first-step screening tool that could be used to identify potential synergies between secondary metabolites, which could be further complemented with chemical analyses to gain more insight into specific mechanisms.

Even though it is difficult to disentangle the positive effects of resource competition and direct pathogen inhibition for the invasion resistance based on our data, structural equation modeling suggests that both modes of competition played significant roles. In particular, the niche breadth of the introduced *Pseudomonas* community was important by increasing the *Pseudomonas* density and decreasing the pathogen density. However, fewer clear patterns were found in the case of disease incidence, where only the *Pseudomonas* community richness seemed to significantly reduce disease development. This suggests that the high *Pseudomonas* community diversity increased plant pathogen suppression via some unidentified function. One such potential function could be bacterial cooperation (15) or facilitation (28). For example, it has been shown that bacteria that adapt to each other in diverse communities become more productive but also more dependent on each other (28). *Pseudomonas* strains are also known to cooperate via production of siderophores that scavenge iron from the environment (6, 28). The extent to which these positive interactions affected the survival and the invasion resistance of the most diverse *Pseudomonas* communities in the present study is unknown. Moreover, bacterial diversity may also affect traits, such as biofilm formation or stress resistance, which are not captured in the measured parameters but may be important for function in the rhizosphere environment. This may explain why richness, but not the traits from the laboratory assays, predicted tomato disease. Regardless of these potential limitations, our data suggest that biodiversity-ecosystem functioning relationships are good indicators of the benefits of plant growth-promoting bacterial communities for host plants.

Interestingly, diversity effects rather than the identity effects drove the functioning of the *Pseudomonas* communities once introduced into the natural rhizosphere microbiome: all strains grown in mixed communities performed better than monocultures, and the invasion resistance was not systematically improved by the inclusion of any particular *Pseudomonas* strain. This suggests that pathogen suppression was an emergent and diversity-dependent community-level property. These findings have important implications for applied biology. Synthetic microbial communities are widely used in biotechnological processes due to their ability to provide functional properties that a single microbial species or strain cannot offer (29–31). Our findings suggest that biodiversity-ecosystem functioning theory can guide assembly of effective bacterial communities that reliably enhance microbiome function. We suggest that the present community assembly principles can be transferred to other fields of microbiome research and biotechnology due to the presence of very general ecological mechanisms. Creating functionally diverse microbial consortia may increase the provisioning of focal functions, particularly in complex environments, such as the rhizosphere (32). Assemblages of different microorganisms combine properties unattainable by a single strain or species (29, 33, 34) and have been proposed as a solution to improve industrial and agronomic processes (31, 35, 36).

## MATERIALS AND METHODS

### Bacterial study strains.

We used eight fluorescent pseudomonad strains (CHA0, PF5, Q2-87, Q8R1-96, 1M1-96, MVP1-4, F113, and Phl1C2) as described previously (20) (for more information, see Table S2 in the supplemental material). All strains were stored at −80°C. Prior to the experiments, a single colony of each strain was selected randomly, grown overnight in lysogenic broth (LB), washed three times in 0.85% NaCl, and adjusted to an optical density at 600 nm (OD_600_) of 0.5 using a spectrophotometer (Spectra Max M5; Molecular Devices, Sunnyvale, CA). We used *Ralstonia solanacearum* strain QL-Rs1115 (race 1 and biovar 3) as a pathogen. This strain was originally isolated from a tomato rhizosphere in Qilin (118°57′E, 32°03′N), Nanjing, China, is highly virulent, and is able to cause wilting of tomato, eggplant, pepper, and potato (13).

### Assembly of *Pseudomonas* communities.

We created 48 communities by the use of eight different *Pseudomonas* strains, which we combined following a substitutive design as described previously (19) to obtain initial richness levels of 1, 2, 4, and 8 strains (Table S3). The diversity gradient was assembled so that each strain was drawn randomly, allowing disentangling the effects of strain identity and community diversity. We used a substitutive design so that the total biomass of every *Pseudomonas* community inoculant was kept the same in all treatments but the proportion of every single strain decreased with increasing community richness (100%, 50%, 25%, and 12.5% for 1-, 2-, 4-, and 8-strain communities, respectively).

### Characterizing BEF relationships *in vitro*.

In order to link biodiversity effects to bacterial resource competition, we assessed the resource use of the eight *Pseudomonas species* and *R. solanacearum* strains on 48 different single-carbon resources (Table S4) representative of tomato root exudates (5). Briefly, bacteria grown overnight in tryptic soy broth (TSB; tryptone at 15 g liter^−1^, soy peptone at 5 g liter^−1^, NaCl at 5 g liter^−1^) were pelleted by centrifugation (4,000 × *g*, 3 min) and washed three times in 0.85% NaCl before their growth was measured on 96-well microtiter plates containing Os minimal medium (37) supplemented with a 10 mM concentration of a single resource representative of amino acids, organic acids, and sugars found in tomato root exudates (5). We used a total of 48 different single compounds as listed in Table S4. All microplate wells were inoculated with equal amounts of the specified bacterial mixtures (starting OD_600_ = 0.05) and incubated for 48 h with agitation (170 rpm) at 30°C. Optical density (600 nm) was recorded at regular intervals with a spectrophotometer (Spectra Max M5; Molecular Devices, Sunnyvale, CA). Community-level resource use metrics were characterized using two indices, the niche breadth index and the niche overlap index, defined as the number of resources consumed by the *Pseudomonas* communities and the proportion of each resource used by *R. solanacearum* and the *Pseudomonas* community, respectively. Wells with an OD_600_ greater than 0.05 were scored as representing positive growth on any given substrate.

In order to link biodiversity effects to direct inhibition of the pathogen, we quantified the pathogen growth in the presence of *Pseudomonas* supernatants. To avoid biases due to competition or facilitation between different *Pseudomonas* strains, we grew all eight *Pseudomonas* strains individually in nutrient broth for 30 h (30°C, 170 rpm), after which cells were pelleted by centrifugation (4,000 × *g*, 3 min). Cell-free supernatants were then mixed in proportions matching the diversity gradient of the communities (1-, 2-, 4-, and 8-strain richness levels; Table S3), and inhibition experiments were started immediately. Briefly, 20 μl of supernatant mix was added to a fresh culture (180 µl, OD_600_ = 0.05) of the pathogen *R. solanacearum* in modified standard mineral salt agar (M-SMSA) media (38). Control treatments received 20 µl M-SMSA media. Bacteria were grown for 24 h (30°C, 170 rpm) before bacterial densities were measured as optical density at 600 nm using a spectrophotometer (Spectra Max M5 plate reader; Molecular Devices, Sunnyvale, CA). Pathogen inhibition was defined as the percentage of reduction in pathogen growth compared to pathogen growth in the control treatment.

### Validating BEF relationships in a greenhouse experiment.

The biocontrol efficiency of *Pseudomonas* bacterial communities was assessed in a 50-day-long greenhouse experiment (an overview of the protocol is presented in Fig. S1 in the supplemental material). The soil was collected from a tomato field in Qilin, a town of Nanjing, China (13), sieved at 5 mm, and homogenized. Please note that the homogenized soil contained the natural microbial community. We used the same 48 *Pseudomonas* community combinations as were used in the *in vitro* experiments (Table S3). Surface-sterilized tomato seeds (*Lycopersicon esculentum*, cultivar “*Jiangshu*”) were germinated on water-agar plates for 3 days before being sown into seedling plates containing cobalt-60-sterilized seedling substrate (Huainong, Huaian Soil and Fertilizer Institute, Huaian, China). Germinated tomato plants were transplanted to seedling trays containing natural, nonsterile soil at the three-leaf stage of growth (12 days after sowing). Twenty-four seedlings were transplanted into one seedling tray with 8 cells, each of which contained 500 g soil planted with three seedlings. Each tray was treated as one biological replicate. Two replicate seedling plates were used for all communities (and four replicate plates for a positive control). After 10 days of growth, plants were inoculated with *Pseudomonas* communities by the use of root drenching methods with a final concentration of 5.0 × 10^7^ CFU of bacteria g^−1^ soil (39). After 5 days postinoculation of *Pseudomonas* communities, the pathogen *R. solanacearum* was inoculated at a final concentration of 10^6^ CFU of bacteria g^−1^ soil. Tomato plants were then grown for 35 days in a greenhouse (with natural temperature variation ranging from 25°C to 35°C) and watered regularly with sterile water. Disease incidence per seedling plate was used as a disease index (13). Seedling plates were rearranged randomly every 2 days. Disease progression was monitored daily after the pathogen inoculation. The experiment was terminated 35 days after pathogen inoculation when all the plants given the positive-control treatment showed symptoms of wilting.

### Tomato rhizosphere sampling and DNA extraction.

We performed destructive sampling to estimate pathogen and introduced *Pseudomonas* abundances 5, 15, 25, and 35 days after the pathogen inoculation. We removed two randomly chosen plants per community from one of the replicate seedling plates (total of 416 rhizosphere samples) at every time point. Rhizosphere soil was collected by first gently removing the plants from the pots before shaking off excess soil and collecting the soil attached to the roots. Samples were stored at −80°C for DNA extraction. Microbial DNA was extracted using a Power Soil DNA isolation kit (Mo Bio Laboratories, Inc., Carlsbad, CA) following the manufacturer’s protocol. DNA quality was checked by running samples by the use of 1% sodium boric acid agarose gel electrophoresis, and DNA concentrations were determined by using a NanoDrop 1000 spectrophotometer (Thermo Scientific, Waltham, MA). Extracted DNA was stored at −80°C for bacterial density analyses.

### Pathogen and *Pseudomonas* bacterial densities in the rhizosphere.

We used quantitative PCR (qPCR) to quantify the abundance of the introduced *Pseudomonas* bacteria and the pathogen in the rhizosphere soil. *Pseudomonas* bacterial density was estimated with primers B2BF (5′-ACC CAC CGC AGC ATC GTT TAT GAG C-3′) and B2BR3 (5′-AGC AGA GCG ACG AGA ACT CCA GGG A-3′) targeting the *phlD* gene (40), which is part of the *phl* operon responsible for the synthesis of the broad-spectrum antibiotic 2,4-diacetylphloroglucinol (DAPG). We use this gene as a reference because it is shared by all of the *Pseudomonas* strains that we used while being present at only a low background concentration in the reference soil (the background level is shown in all figures as a red dashed line). Pathogen density was quantified by using specific primers (forward, 5′-GAA CGC CAA CGG TGC GAA CT-3′; reverse, 5′-GGC GGC CTT CAG GGA GGT C-3′) targeting the *fliC* gene coding the flagellum subunit (41). The qPCR analyses were carried out with an Applied Biosystems 7500 real-time PCR system (Applied Biosystems, CA) using SYBR green I fluorescent dye detection in 20-μl volumes containing 10 μl of SYBR Premix *Ex Taq* (TaKaRa Bio Inc., Japan), 2 μl of template, and 0.4 μl of both forward and reverse primers (10 mM each). The PCR was performed by initially denaturizing at 95°C for 30 s, cycling 40 times with a 5-s denaturizing step at 95°C following a 34-s elongation/extension step at 60°C, and ending with melt curve analysis at 95°C for 15 s, at 60°C for 1 min, and at 95°C for 15 s. Each sample experiment was replicated three times.

### Statistical analyses.

For *in vitro* experiments, we used generalized linear models (GLM) to test whether *Pseudomonas* community richness affects niche breadth, niche overlap with the pathogen, and direct pathogen inhibition.

### Greenhouse experiment.

Data were analyzed in three ways. First, we used separate GLMs expressing disease incidence as well as pathogen and *Pseudomonas* community abundances as a function of the interactive effects of time and *Pseudomonas* community richness. Bacterial abundance data were log_10_ transformed and disease incidence data square arcsine-transformed prior to analysis. Second, we attempted to link the dependent variables to changes in the characteristics of the *Pseudomonas* community, including resource competition metrics (niche breadth and niche overlap), direct pathogen inhibition (toxicity), and *Pseudomonas* community density in the rhizosphere. Due to potential correlations between different explanatory variables, a sequential analysis was used to uncover the most parsimonious GLMs. To this end, we used stepwise model selection based on Akaike information criteria (AIC) to choose the model with the best explanatory power [step () function in R]. We used both backward elimination starting with the full model and a forward-selection model (from simple to full model) to avoid selecting a local AIC minimum (42). Finally, we used structural equation modeling (SEM) to shed light on the mechanisms of disease incidence in tomato plants by accounting for multiple potentially correlated effect pathways. SEM analysis was chosen because it can disentangle the direct and indirect effects (43) of diversity and community characteristic parameters *in vitro* for determining the survival of *Pseudomonas* communities, the pathogen density in tomato rhizosphere, and the disease incidence in the greenhouse experiment. The initial model was based on previous knowledge (44) for assigning the exogenous variable “richness” and the endogenous variables “niche breadth,” “niche overlap,” “toxin production,” “*Pseudomonas* density,” “pathogen density,” and “disease incidence.” Due to the relatively low level of replication and the complex structural equation model, we ran separate models for “pathogen density” and “disease incidence.” The adequacy of the models was determined via chi-square tests, AIC, and root mean square error of approximation (RMSEA) (44). Model modification indices and stepwise removal of nonsignificant relationships were used to improve the models; however, only scientifically sound relationships were considered (43). Structural equation modeling was performed using Amos 5 (Amos Development Corporation, Crawfordville, FL).

## SUPPLEMENTAL MATERIAL

Figure S1  Overview of the greenhouse experiment. (A and B) Surface-sterilized tomato seeds (*Lycopersicon esculentum*, cultivar “Jiangshu”) were germinated on water-agar plates for 3 days (A) before sowing into seedling plates containing cobalt-60-sterilized seedling substrate (Huainong, Huaian Soil and Fertilizer Institute, Huaian, China) was performed (B). (C) At the three-leaf stage (12 days after sowing), tomato plants were transplanted to seedling trays (350 mm by 250 mm by 100 mm) containing the same natural soil as that described in Materials and Methods. Sixteen seedlings were transplanted into one seedling tray with 8 cells, with each containing two seedlings. Tomato plants were first inoculated with *Pseudomonas* bacterial communities by the drenching method (13) 10 days after the transplantation (with an ending *Pseudomonas* density of 5.0 × 10^7^ CFU g^−1^ soil). The pathogen was inoculated 5 days later (with an ending *R. solanacearum* density of 10^6^ CFU g^−1^ soil). Tomato plants were grown in a greenhouse with a natural daily temperature variation ranging from 25°C to 35°C and were watered regularly with sterile water. (D) The number of wilted plants per seedling plate was recorded on a daily basis after the pathogen inoculation. Red flags represent the number of wilted and infected tomato plants (E). The experiment was ended 50 days after the transplantation when all the plants in the control treatment (*R. solanacearum* only) showed disease symptoms. Download Figure S1, DOCX file, 2.1 MB

Table S1  Analysis of variance showing the effect of *Pseudomonas* strain identity on disease incidence, pathogen and *Pseudomonas* community abundance, and transgressive overyielding (*Pseudomonas* strain abundances when grown in polycultures versus monocultures) in *Pseudomonas* communities at 5 days, 15 days, 25 days, and 35 days post-pathogen inoculation (dpi). Table S1, DOCX file, 0.03 MB

Table S2  List of the bacterial species and strains used in this study. Table S2, DOCX file, 0.02 MB

Table S3  Composition of the *Pseudomonas* bacterial communities used in this study (0 and 1 denote the absence and presence of *Pseudomonas* strains in a given community, respectively). Table S3, DOCX file, 0.02 MB

Table S4  Carbon resources used to quantify pathogen and *Pseudomonas* community resource use metrics (niche breadth and niche overlap). Table S4, DOCX file, 0.01 MB
