# Supplementary material for: Probiotic Diversity Enhances Rhizosphere Microbiome Function and Plant Disease Suppression
Source: mBio. 2016 Dec 13;7(6):e01790-16. doi: 10.1128/mBio.01790-16 (PMC5156302; doi:10.1128/mBio.01790-16)
Supplement: Figure S1 — Overview of the greenhouse experiment. (A and B) Surface-sterilized tomato seeds (Lycopersicon esculentum, cultivar “Jiangshu”) were germinated on water-agar plates for 3 days (A) before sowing into seedling plates containing cobalt-60-sterilized seedling substrate (Huainong, Huaian Soil and Fertilizer Institute, Huaian, China) was performed (B). (C) At the three-leaf stage (12 days after sowing), tomato plants were transplanted to seedling trays (350 mm by 250 mm by 100 mm) containing the same natural soil as that described in Materials and Methods. Sixteen seedlings were transplanted into one seedling tray with 8 cells, with each containing two seedlings. Tomato plants were first inoculated with Pseudomonas bacterial communities by the drenching method (13) 10 days after the transplantation (with an ending Pseudomonas density of 5.0 × 10^7^ CFU g−1 soil). The pathogen was inoculated 5 days later (with an ending R. solanacearum density of 106 CFU g−1 soil). Tomato plants were grown in a greenhouse with a natural daily temperature variation ranging from 25°C to 35°C and were watered regularly with sterile water. (D) The number of wilted plants per seedling plate was recorded on a daily basis after the pathogen inoculation. Red flags represent the number of wilted and infected tomato plants (E). The experiment was ended 50 days after the transplantation when all the plants in the control treatment (R. solanacearum only) showed disease symptoms. Download [file mbo006163108sf1.docx]

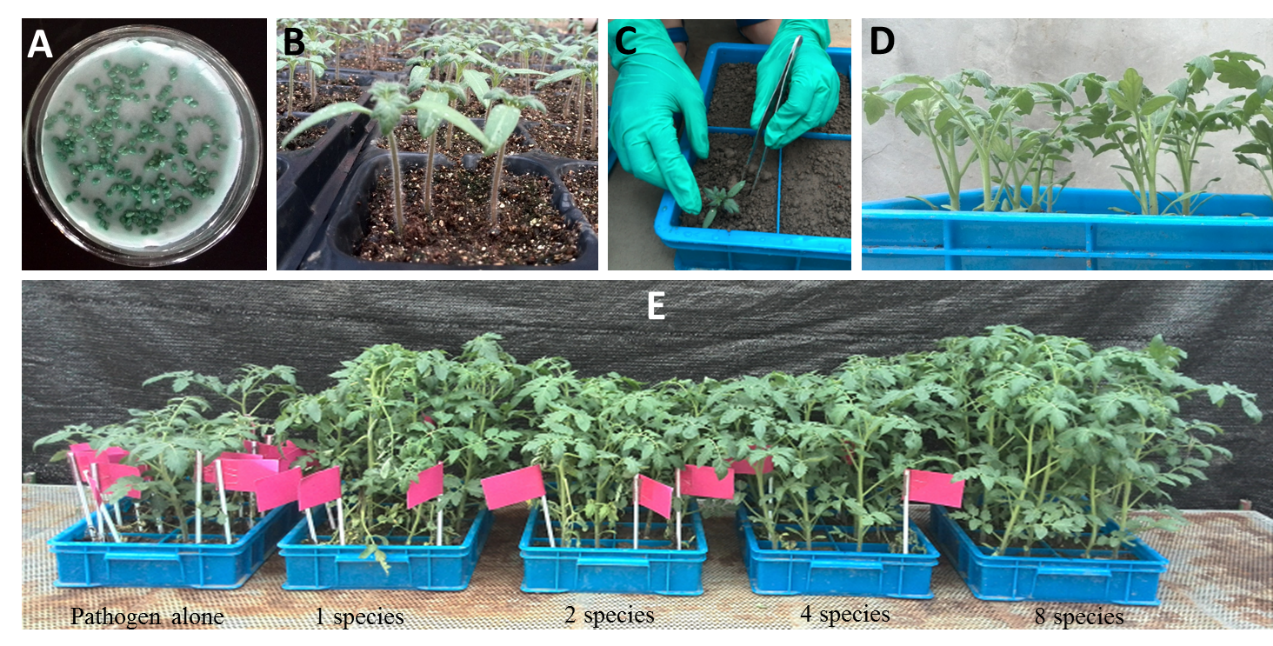


Figure S1. Overview of the greenhouse experiment. Surface-sterilized tomato seeds (*Lycopersicon esculentum*, cultivar “Jiangshu”) were germinated on water-agar plates for three days (A) before sowing into seedling plates (B) containing Cobalt -60-sterilized seedling substrate (Huainong, Huaian soil and fertilizer Institute, Huaian, China). At the three-leaf stage (12 days after sowing), tomato plants were transplanted to seedling trays (350mm×250mm×100mm) containing the same natural soil as described in the materials and methods (C). Sixteen seedlings were transplanted into one seedling tray with 8 cells with each containing two seedlings. Tomato plants were first inoculated with *Pseudomonas* bacterial communities by drenching method (Wei et al. 2011) ten days after the transplantation (with ending *Pseudomonas* density of 5.0 × 10^7^ CFU g ^-1^ soil). Pathogen was inoculated five days later (ending *R. solanacearum* density of 10^6^ CFU g^-1^ soil). Tomato plants were grown in a greenhouse with natural daily temperature variation ranging from 25 °C to 35 °C and watered regularly with sterile water. The number of wilted plants per seedling plate was recorded on daily basis after the pathogen inoculation (D-E): red flags represent the number of wilted and infected tomato plants. The experiment was ended 50 days after the transplantation when all the plants in the control treatment (*R. solanacearum* only) showed disease symptoms.
