# Supplementary material for: Probiotic Diversity Enhances Rhizosphere Microbiome Function and Plant Disease Suppression
Source: mBio. 2016 Dec 13;7(6):e01790-16. doi: 10.1128/mBio.01790-16 (PMC5156302; doi:10.1128/mBio.01790-16)
Supplement: Table S1 — Analysis of variance showing the effect of Pseudomonas strain identity on disease incidence, pathogen and Pseudomonas community abundance, and transgressive overyielding (Pseudomonas strain abundances when grown in polycultures versus monocultures) in Pseudomonas communities at 5 days, 15 days, 25 days, and 35 days post-pathogen inoculation (dpi). [file mbo006163108st1.docx]

Table S1. Analysis of variance showing the effect of *Pseudomonas* strains’ identity on disease incidence, pathogen and *Pseudomonas* community abundance, and transgressive overyielding (*Pseudomonas* strain abundances when grown in polycultures versus monocultures) in *Pseudomonas* communities at 5 days, 15 days, 25 days and 35 days post pathogen inoculation (dpi).

|  |  | Disease Incidence | | | | | | | | | | |
| --- | --- | --- | --- | --- | --- | --- | --- | --- | --- | --- | --- | --- |
|  |  | 5 dpi | | | 15 dpi | | | 25 dpi | | | 35 dpi | |
| *Pseudomonas* strains | df | F | P | | F | P | | F | | P | F | P |
| MVP1-4 | 1 | - | - | | 0.3 | 0.5977 | | 2.4 | | 0.1329 | 1.7 | 0.1985 |
| Q2-87 | 1 | - | - | | 0.1 | 0.8190 | | 0.4 | | 0.5220 | 0.0 | 0.9891 |
| CHA0 | 1 | - | - | | 0.7 | 0.4079 | | 0.4 | | 0.5133 | 0.9 | 0.3550 |
| F113 | 1 | - | - | | 2.1 | 0.1596 | | 0.0 | | 0.8384 | 0.0 | 0.9238 |
| Phl1c2 | 1 | - | - | | 1.1 | 0.2940 | | 1.9 | | 0.1777 | 2.1 | 0.1533 |
| PF5 | 1 | - | - | | 0.0 | 0.8545 | | 0.0 | | 0.8467 | 0.3 | 0.5666 |
| 1M1-96 | 1 | - | - | | 0.1 | 0.8180 | | 0.2 | | 0.6473 | 0.1 | 0.8213 |
| Q8R1-96 | 1 | - | - | | 0.0 | 0.9791 | | 0.0 | | 0.9583 | 0.1 | 0.7892 |
| Residuals | 40 |  |  | |  |  | |  | |  |  |  |
| log(richness) | 1 |  |  | | 4.5 | 0.0395 | | 67.4 | | <0.0001 | 150.2 | <0.0001 |
| Residuals | 46 |  |  | |  |  | |  | |  |  |  |
|  |  | Pathogen abundance | | | | | | | | | | |
| MVP1-4 | 1 | 1.1 | 0.2939 | | 1.2 | 0.2710 | | 1.0 | | 0.3277 | 0.7 | 0.4137 |
| Q2-87 | 1 | 1.3 | 0.2628 | | 1.3 | 0.2564 | | 1.1 | | 0.3088 | 1.1 | 0.3117 |
| CHA0 | 1 | 1.3 | 0.2619 | | 1.3 | 0.2568 | | 1.2 | | 0.2763 | 1.3 | 0.2522 |
| F113 | 1 | 1.2 | 0.2802 | | 1.1 | 0.3056 | | 1.0 | | 0.3245 | 0.8 | 0.3699 |
| Phl1c2 | 1 | 1.3 | 0.2567 | | 1.7 | 0.1964 | | 1.2 | | 0.2872 | 1.4 | 0.2417 |
| PF5 | 1 | 1.4 | 0.2504 | | 1.3 | 0.2618 | | 0.9 | | 0.3526 | 0.7 | 0.3972 |
| X1M1-96 | 1 | 1.1 | 0.2928 | | 0.9 | 0.3606 | | 0.8 | | 0.3689 | 0.7 | 0.4069 |
| Q8R1-96 | 1 | 1.2 | 0.2784 | | 0.8 | 0.3643 | | 0.9 | | 0.3473 | 0.9 | 0.3492 |
| Residuals | 40 |  |  | |  |  | |  | |  |  |  |
| log(richness) | 1 | 381.5 | <0.0001 | | 381.5 | <0.0001 | | 559.0 | | <0.0001 | 465.9 | <0.0001 |
| Residuals | 46 |  |  | |  |  | |  | |  |  |  |
|  |  | *Pseudomonas* abundance | | | | | | | | | | |
| MVP1-4 | 1 | 1.3 | 0.2659 | | 1.1 | 0.3037 | | 1.7 | | 0.2053 | 1.6 | 0.2071 |
| Q2-87 | 1 | 1.8 | 0.1855 | | 1.7 | 0.2042 | | 2.1 | | 0.1515 | 1.3 | 0.2653 |
| CHA0 | 1 | 0.8 | 0.3723 | | 1.3 | 0.2607 | | 1.1 | | 0.2937 | 1.2 | 0.2842 |
| F113 | 1 | 1.7 | 0.2017 | | 1.4 | 0.2393 | | 1.3 | | 0.2640 | 1.9 | 0.1731 |
| Phl1c2 | 1 | 1.2 | 0.2897 | | 1.2 | 0.2746 | | 0.9 | | 0.3459 | 1.4 | 0.2380 |
| PF5 | 1 | 1.0 | 0.3327 | | 1.5 | 0.2251 | | 1.2 | | 0.2751 | 1.4 | 0.2420 |
| 1M1-96 | 1 | 1.9 | 0.1783 | | 1.5 | 0.2208 | | 1.2 | | 0.2821 | 1.2 | 0.2847 |
| Q8R1-96 | 1 | 0.9 | 0.3494 | | 1.4 | 0.2464 | | 1.6 | | 0.2080 | 1.6 | 0.2066 |
| Residuals | 40 |  |  | |  |  | |  | |  |  |  |
| log(richness) | 1 | 326.1 | <0.0001 | | 289.5 | <0.0001 | | 414.1 | | <0.0001 | 342.8 | <0.0001 |
| Residuals | 46 |  |  | |  |  | |  | |  |  |  |
|  |  | Transgressive overyielding in *Pseudomonas* communities | | | | | | | | | | |
| MVP1-4 | 1 | 0.2 | | 0.6497 | 0.7 | | 0.4118 | | 0.4 | 0.5322 | 2.2 | 0.1442 |
| Q2-87 | 1 | 0.1 | | 0.7912 | 0.3 | | 0.6060 | | 0.0 | 0.8353 | 0.6 | 0.4416 |
| CHA0 | 1 | 0.2 | | 0.6325 | 0.7 | | 0.4121 | | 0.0 | 0.9064 | 0.4 | 0.5199 |
| F113 | 1 | 1.9 | | 0.1802 | 8.7 | | 0.0053 | | 1.4 | 0.2496 | 1.1 | 0.3031 |
| Phl1c2 | 1 | 0.3 | | 0.5585 | 0.0 | | 0.9914 | | 0.2 | 0.7002 | 1.2 | 0.2792 |
| PF5 | 1 | 0.5 | | 0.4989 | 1.5 | | 0.2226 | | 0.8 | 0.3640 | 0.0 | 0.8364 |
| 1M1-96 | 1 | 0.6 | | 0.4492 | 0.0 | | 0.9493 | | 0.0 | 0.9129 | 0.3 | 0.5606 |
| Q8R1-96 | 1 | 0.7 | | 0.4196 | 4.0 | | 0.0522 | | 0.0 | 0.9293 | 3.4 | 0.0720 |
| Residuals | 40 |  | |  |  | |  | |  |  |  |  |
| log(richness) | 1 | 0.3 | | 0.5712 | 2.7 | | 0.1101 | | 0.6 | 0.4267 | 10.4 | 0.0023 |
| Residuals | 46 |  | |  |  | |  | |  |  |  |  |
