# Supplementary material for: Probiotic Diversity Enhances Rhizosphere Microbiome Function and Plant Disease Suppression
Source: mBio. 2016 Dec 13;7(6):e01790-16. doi: 10.1128/mBio.01790-16 (PMC5156302; doi:10.1128/mBio.01790-16)
Supplement: Table S2 — List of the bacterial species and strains used in this study. [file mbo006163108st2.docx]

Table S2. List of the *Pseudomonas* strains used in this study

| Organisms | Origin | Reference |
| --- | --- | --- |
| ***Pseudomonas* strains** |  |  |
| *Pseudomonas protegens* CHA0 | Tobacco, Switzerland | (Natsch A, Keel C, Pfirter HA, Haas D, Defago G.,Appl Environ Microbiol 60:2553-2560, 1994) |
| *Pseudomonas fluorescens* F113 | Sugar beet, Irland | (Shanahan P, Osullivan DJ, Simpson P, Glennon JD, Ogara F. ,Appl Environ Microbiol 58:353-358, 1992) |
| *Pseudomonas fluorescens* Phl1C2 | Tomato, France | (De La Fuente L, Mavrodi DV, Landa BB, Thomashow LS, Weller DM. FEMS Microbiol Ecol 56:64-78, 2006) |
| *Pseudomonas protegens*  Pf-5 | Cotton, USA | (Howell CR, Stipanovic RD. Phytopathology 69:480-482,1979) |
| *Pseudomonas fluorescens* Q2-87 | Wheat, USA | (Bangera MG, Thomashow LS., J Bacteriol 181:3155-3163,1999) |
| *Pseudomonas brassicacearum* Q8R1-96 | Wheat, USA | (Raaijmakers JM, Weller DM., Mol Plant Microbe Interact 11:144-152,1998) |
| *Pseudomonas fluorescens* 1M1-96 | Wheat, USA | (Raaijmakers JM, Weller DM. Appl Environ Microbiol 67:2545-2554,2001) |
| *Pseudomonas fluorescens* MVP1-4 | Pea, USA | (Landa BB, Mavrodi OV, Raaijmakers JM, Gardener BBM, Thomashow LS, Weller DM. Appl Environ Microbiol 68:3226-3237,2002) |
| **Pathogen** |  |  |
| *Ralstonia solanacearum* QL-Rs1115 | Tomato, China | (Wei Z, Yang X, Yin S, Shen Q, Ran W, Xu Y. APPL SOIL ECOL 48:152-159,2011) |

Note: See also (Loper JE, Hassan KA, Mavrodi DV, Davis EW, 2nd, Lim CK, Shaffer BT, Elbourne LD, Stockwell VO, Hartney SL, Breakwell K, Henkels MD, Tetu SG, Rangel LI, Kidarsa TA, Wilson NL, van de Mortel JE, Song C, Blumhagen R, Radune D, Hostetler JB, Brinkac LM, Durkin AS, Kluepfel DA, Wechter WP, Anderson AJ, Kim YC, Pierson LS, 3rd, Pierson EA, Lindow SE, Kobayashi DY, Raaijmakers JM, Weller DM, Thomashow LS, Allen AE, Paulsen IT., vol 8, p e1002784,2012 )for an updated classiﬁcation of the Pseudomonas strains; + toxin production, DAPG 2, 4-diacetylphloroglucinol, PLT pyoluteorin, PRN pyrrolnitrin, HCN hydrogen cyanide, AprA extracellular protease.
