# Supplementary material for: Probiotic Diversity Enhances Rhizosphere Microbiome Function and Plant Disease Suppression
Source: mBio. 2016 Dec 13;7(6):e01790-16. doi: 10.1128/mBio.01790-16 (PMC5156302; doi:10.1128/mBio.01790-16)
Supplement: Table S3 — Composition of the Pseudomonas bacterial communities used in this study (0 and 1 denote the absence and presence of Pseudomonas strains in a given community, respectively). [file mbo006163108st3.docx]

Table S3. Composition of the *Pseudomonas* bacterial communities used in this study (0 and 1 denote for the absence and presence of *Pseudomonas* strains in given community, respectively).

| Communities/ *Pseudomonas* strains | MVP1-4 | Q2-87 | CHA0 | F113 | Phl1c2 | PF5 | 1M1-96 | Q8R1-96 | Richness |
| --- | --- | --- | --- | --- | --- | --- | --- | --- | --- |
| 1 | 1 | 0 | 0 | 0 | 0 | 0 | 0 | 0 | 1 |
| 2 | 0 | 1 | 0 | 0 | 0 | 0 | 0 | 0 | 1 |
| 3 | 0 | 0 | 1 | 0 | 0 | 0 | 0 | 0 | 1 |
| 4 | 0 | 0 | 0 | 1 | 0 | 0 | 0 | 0 | 1 |
| 5 | 0 | 0 | 0 | 0 | 1 | 0 | 0 | 0 | 1 |
| 6 | 0 | 0 | 0 | 0 | 0 | 1 | 0 | 0 | 1 |
| 7 | 0 | 0 | 0 | 0 | 0 | 0 | 1 | 0 | 1 |
| 8 | 0 | 0 | 0 | 0 | 0 | 0 | 0 | 1 | 1 |
| 9 | 1 | 0 | 0 | 0 | 0 | 0 | 0 | 0 | 1 |
| 10 | 0 | 1 | 0 | 0 | 0 | 0 | 0 | 0 | 1 |
| 11 | 0 | 0 | 1 | 0 | 0 | 0 | 0 | 0 | 1 |
| 12 | 0 | 0 | 0 | 1 | 0 | 0 | 0 | 0 | 1 |
| 13 | 0 | 0 | 0 | 0 | 1 | 0 | 0 | 0 | 1 |
| 14 | 0 | 0 | 0 | 0 | 0 | 1 | 0 | 0 | 1 |
| 15 | 0 | 0 | 0 | 0 | 0 | 0 | 1 | 0 | 1 |
| 16 | 0 | 0 | 0 | 0 | 0 | 0 | 0 | 1 | 1 |
| 17 | 1 | 1 | 0 | 0 | 0 | 0 | 0 | 0 | 2 |
| 18 | 0 | 1 | 1 | 0 | 0 | 0 | 0 | 0 | 2 |
| 19 | 1 | 0 | 0 | 1 | 0 | 0 | 0 | 0 | 2 |
| 20 | 0 | 1 | 0 | 0 | 1 | 0 | 0 | 0 | 2 |
| 21 | 1 | 0 | 0 | 0 | 0 | 0 | 1 | 0 | 2 |
| 22 | 0 | 0 | 1 | 1 | 0 | 0 | 0 | 0 | 2 |
| 23 | 1 | 0 | 0 | 0 | 0 | 0 | 0 | 1 | 2 |
| 24 | 0 | 0 | 1 | 0 | 0 | 1 | 0 | 0 | 2 |
| 25 | 0 | 0 | 0 | 1 | 1 | 0 | 0 | 0 | 2 |
| 26 | 0 | 0 | 1 | 0 | 0 | 0 | 1 | 0 | 2 |
| 27 | 0 | 1 | 0 | 0 | 0 | 1 | 0 | 0 | 2 |
| 28 | 0 | 0 | 0 | 1 | 0 | 0 | 0 | 1 | 2 |
| 29 | 0 | 0 | 0 | 0 | 1 | 0 | 0 | 1 | 2 |
| 30 | 0 | 0 | 0 | 0 | 1 | 1 | 0 | 0 | 2 |
| 31 | 0 | 0 | 0 | 0 | 0 | 1 | 1 | 0 | 2 |
| 32 | 0 | 0 | 0 | 0 | 0 | 0 | 1 | 1 | 2 |
| 33 | 1 | 1 | 0 | 1 | 1 | 0 | 0 | 0 | 4 |
| 34 | 0 | 1 | 0 | 1 | 0 | 1 | 0 | 1 | 4 |
| 35 | 1 | 0 | 0 | 1 | 1 | 1 | 0 | 0 | 4 |
| 36 | 0 | 1 | 0 | 0 | 1 | 0 | 1 | 1 | 4 |
| 37 | 1 | 1 | 1 | 1 | 0 | 0 | 0 | 0 | 4 |
| 38 | 0 | 1 | 0 | 0 | 0 | 1 | 1 | 1 | 4 |
| 39 | 1 | 0 | 0 | 0 | 1 | 1 | 1 | 0 | 4 |
| 40 | 0 | 1 | 1 | 0 | 1 | 0 | 0 | 1 | 4 |
| 41 | 1 | 0 | 1 | 0 | 0 | 1 | 1 | 0 | 4 |
| 42 | 0 | 0 | 1 | 1 | 0 | 1 | 0 | 1 | 4 |
| 43 | 1 | 0 | 1 | 1 | 0 | 0 | 1 | 0 | 4 |
| 44 | 0 | 0 | 1 | 0 | 1 | 0 | 1 | 1 | 4 |
| 45 | 1 | 1 | 1 | 1 | 1 | 1 | 1 | 1 | 8 |
| 46 | 1 | 1 | 1 | 1 | 1 | 1 | 1 | 1 | 8 |
| 47 | 1 | 1 | 1 | 1 | 1 | 1 | 1 | 1 | 8 |
| 48 | 1 | 1 | 1 | 1 | 1 | 1 | 1 | 1 | 8 |

All *Pseudomonas* monocultures were replicated twice and 8-species communities were replicated for four times. In other richness levels, each probiotic species was included equally often in assembled communities in the same number of communities at each richness level.
