# Supplementary material for: Probiotic Diversity Enhances Rhizosphere Microbiome Function and Plant Disease Suppression
Source: mBio. 2016 Dec 13;7(6):e01790-16. doi: 10.1128/mBio.01790-16 (PMC5156302; doi:10.1128/mBio.01790-16)
Supplement: Table S4 — Carbon resources used to quantify pathogen and Pseudomonas community resource use metrics (niche breadth and niche overlap). [file mbo006163108st4.docx]

Table S4. Carbon resources used to quantify pathogen and *Pseudomonas* community resource use metrics (niche breadth and niche overlap).

| Number | Carbon Source | Number | Carbon Source |
| --- | --- | --- | --- |
| 1 | Acetic aicd | 25 | Malic acid |
| 2 | L-Alanine | 26 | Malonic acid |
| 3 | β-Alanine | 27 | L-Methionine |
| 4 | L-Arginine | 28 | Myoinositol |
| 5 | Ascorbic acid | 29 | 2-Oxoglutaric |
| 6 | L-Asparagine | 30 | L-Phenyalanine |
| 7 | γ-Aminobutyric acid | 31 | L-Proline |
| 8 | Citric acid | 32 | Pyruvic acid |
| 9 | Citrulline | 33 | L-Serine |
| 10 | Ethanolamine | 34 | Succinic acid |
| 11 | Formic acid | 35 | Sucrose |
| 12 | Fructose | 36 | Tartaric acid |
| 13 | Galacturonic acid | 37 | L-Threonine |
| 14 | Glucose | 38 | L-Tryptophan |
| 15 | L-Glutamine | 39 | L-Valine |
| 16 | Glutaric acid | 40 | Maltose |
| 17 | L-Glycine | 41 | L-arabinose |
| 18 | Glycolic acid | 42 | D-galactose |
| 19 | L-Histidine | 43 | D-mannose |
| 20 | Isoleucine | 44 | D-xylose |
| 21 | Lactic acid | 45 | D-ribose |
| 22 | L-Lysine | 46 | D-mannitol |
| 23 | L-Leucine | 47 | Inosine |
| 24 | Maleic acid | 48 | Oxalic acid |
